# A *Porphyromonas gingivalis* Capsule-Conjugate Vaccine Protects From Experimental Oral Bone Loss

**DOI:** 10.3389/froh.2021.686402

**Published:** 2021-07-05

**Authors:** Fernanda G. Rocha, Aym Berges, Angie Sedra, Shirin Ghods, Neeraj Kapoor, Lucy Pill, Mary Ellen Davey, Jeff Fairman, Frank C. Gibson

**Affiliations:** ^1^Department of Oral Biology, University of Florida College of Dentistry, Gainesville, FL, United States; ^2^Vaxcyte Inc., Foster City, CA, United States

**Keywords:** conjugate vaccine, periodontal disease, *Porphyromonas gingivalis*, capsular polysaccharide, oral bone loss

## Abstract

Periodontal diseases are chronic inflammatory diseases of the periodontium that result in progressive destruction of the soft and hard tissues supporting the teeth, and it is the most common cause of tooth loss among adults. In the US alone, over 100 million individuals are estimated to have periodontal disease. Subgingival bacteria initiate and sustain inflammation, and, although several bacteria have been associated with periodontitis, *Porphyromonas gingivalis* has emerged as the key etiological organism significantly contributing to the disease. Currently, intensive clinical maintenance strategies are deployed to mitigate the further progression of disease in afflicted individuals; however, these treatments often fail to stop disease progression, and, as such, the development of an effective vaccine for periodontal disease is highly desirable. We generated a conjugate vaccine, comprising of the purified capsular polysaccharide of *P. gingivalis* conjugated to eCRM®, a proprietary and enhanced version of the CRM197 carrier protein with predetermined conjugation sites (Pg-CV). Mice immunized with alum adjuvanted Pg-CV developed robust serum levels of whole organism-specific IgG in comparison to animals immunized with unconjugated capsular polysaccharide alone. Using the murine oral bone loss model, we observed that mice immunized with the capsule-conjugate vaccine were significantly protected from the effects of *P. gingivalis*-elicited oral bone loss. Employing a preclinical model of infection-elicited oral bone loss, our data support that a conjugate vaccine incorporating capsular polysaccharide antigen is effective in reducing the main clinical endpoint of periodontal disease—oral bone destruction. Further development of a *P. gingivalis* capsule-based conjugate vaccine for preventing periodontal diseases is supported.

## Introduction

Periodontal disease is a chronic oral inflammatory disease that is initiated by a changed subgingival microflora, which leads to the progressive destruction of the soft and hard tissues supporting the teeth and, in severe disease, is the number one cause of tooth loss in adults [1]. In the US, it is estimated that ~42% of the adult population have periodontal disease, thus making this disease one of the most common chronic infectious diseases affecting humans [2]. The economic burden of loss of productivity due to periodontitis was estimated to be $54 billion in 2013 [3]. Although periodontitis is prevalent in adults, it can affect children and adolescents, and is widespread among elderly populations. Periodontitis has also been associated with systemic conditions, such as diabetes, cardiovascular diseases, rheumatoid arthritis, orodigestive cancers, and Alzheimer's disease [4–7]. Current clinical treatment regimens are aggressive and are aimed at halting the progression of the disease to preserve remaining periodontal bone levels; however, despite intensive clinical intervention, failure to halt periodontal disease progression in individuals is often encountered. As such, a vaccine for this disease is highly desirable. Although the etiology of periodontal disease is polymicrobial, *Porphyromonas gingivalis* is among the more commonly encountered organisms known to become overpopulated during the transition to disease [8] and, thus, is identified as key bacteria associated with this disease.

*P. gingivalis* is a Gram-negative anaerobic bacterium that possesses an array of molecules, including fimbriae, two types of lipopolysaccharide, gingipains, and a polysaccharide capsule that contribute to the overall virulence potential of this organism [9–15]. Several groups have targeted these molecules as vaccine candidates and have had varying degrees of success [16–21]. It is known that *P. gingivalis* strains display a broad range of heterogeneity; however, encapsulated strains are associated with highly invasive infection [9, 22]. There appear to be at least 6 serogroups of *P. gingivalis* based on capsular polysaccharide (CPS) serologic characterization [23]. The genetic locus for capsular biosynthesis has been identified [24, 25], and several groups have reported composition and basic structural analysis of the *P. gingivalis* CPS [26, 27]. Previously, it was shown that the CPS of *P. gingivalis* provides a measure of protection to the organism by limiting response to the seen capsulated organisms compared with an isogenic mutant [9]. Interestingly, in pure form, the CPS of this organism has been shown to elicit inflammatory responses from immune cells [28]. Our group and others have focused on *P. gingivalis* CPS as a vaccine, and we have found that mice immunized with this molecule in the purified form are protected from subsequent live organism-elicited oral bone loss [20, 29].

Although there are some examples of polysaccharide vaccines, which evoke T cell response, such as the capsular polysaccharide of *Bacteroides fragilis* [30], bacterial capsular polysaccharides generally only elicit B-cell response and are generally described as T cell-independent antigens [31]. Even though antibodies are produced against the polysaccharide, there is no long-term immune memory, and repeat doses are not typically beneficial, often resulting in lower antibody concentrations compared with single-dose immunizations [32]. As such, the development of efficacious vaccines against polysaccharide-encapsulated pathogens is challenging but can be overcome through the use of polysaccharide-conjugate vaccines, where the polysaccharide is covalently attached to a carrier protein such as tetanus toxoid, *H. influenzae* protein D, or diphtheria toxoid (CRM197) [33]. Polysaccharide-conjugate vaccines have been shown to have enhanced immunogenicity over polysaccharide alone, resulting in a robust immune response across all age groups, and have provided valuable inroads into the development of conjugate vaccines for the treatment of some infectious diseases [34, 35].

The proprietary carrier protein (eCRM®) developed by Vaxcyte, Inc., is an enhanced amino acid variant of the detoxified diphtheria carrier protein CRM197 that retains the glycine to glutamic acid mutation at position 52, which is the basis for the lack of toxicity [36] and which is also constructed with predetermined conjugation sites. CRM197 has a well-established safety profile and has been extensively utilized as the carrier protein in conjugate vaccines for *Streptococcus pneumoniae, Haemophilus influenza type b* (Hib), and *Neisseria meningitides* [37]. Vaxcyte, Inc., uses a cell-free protein synthesis platform to site-selectively insert non-native amino acids (nnAAs) into eCRM®, which then acts as the carrier protein for a conjugate vaccine. This allows for site-specific conjugation of the capsular polysaccharide, using copper-free-click chemistry in order to produce the final polysaccharide-protein conjugate. The traditional version of the carrier protein, CRM197, contains 39 lysine amino acid residues, of which ~20% are located on the protein surface and border, or are present in currently identified human T-cell epitopes [38–40]. The use of eCRM® and conjugation via copper-free click chemistry represents an advance over conventional conjugation chemistry that is haphazard where the polysaccharide antigen is allowed to react with any surface-exposed lysine residue and, as such, covalent attachment to the carrier at a lysine residue that is within a human T-cell epitope and thus could block the presentation of the T-cell epitope to the immune system, preventing the induction of long-lasting immune responses.

The aim of the present study was to determine the efficacy of a novel polysaccharide-peptide conjugate vaccine based on the CPS of *P. gingivalis* conjugated to eCRM® (Pg-CV) in the prevention of oral bone loss, using a murine preclinical model of oral infection. We observed that, in comparison to control animals and mice orally challenged with *P. gingivalis* only, mice immunized with the Pg-CV developed a robust IgG response directed at whole bacteria. Importantly, mice immunized with Pg-CV were protected from oral bone loss. The data are promising to support the development of a capsular polysaccharide-based conjugate vaccine as a potential novel therapeutic vaccine for use in the treatment of periodontal disease.

## Methods

### Cultivation of *P. gingivalis*

For all studies, we used *P. gingivalis* strain A7436, grown anaerobically as previously described [21]. In brief, bacteria were plated from freezer stocks on brain-heart infusion blood agar supplemented with yeast extract, and harvested plate growth was then cultured in BHI-YE broth to a late log phase. For capsule preparations, 2L of broth-cultivated bacteria were frozen, disrupted, washed three times with saline, spun to collect wet cell pellets, and frozen at −80°C until treated with hot phenol to extract capsular polysaccharide as described below. In oral bone loss experiments, overnight cultures of BHI-YE broth-grown *P. gingivalis* were harvested, washed three times with pyrogen-free saline, and suspended to OD_660_ of 1.0, following a 10-fold concentration. In separate experiments, washed and formaldehyde-fixed cultures of broth-grown *P. gingivalis* were used to coat wells of 96-well-plates for ELISA assays.

### Cell Harvest, Hot Phenol-Water/Ether Extraction, and Purification of Capsular Polysaccharide

The *P. gingivalis* CPS was extracted, using a hot-phenol/ether-water method essentially as described previously [20, 26, 41, 42], with some differences. Briefly, frozen *P. gingivalis* cell pellets were treated with hot-phenol water; the aqueous phase was collected and underwent two ether extractions to remove any residual phenol. This crude CPS was then dialyzed extensively and stored at −80°C. As needed, frozen aqueous crude extracts were thawed, enzymatically treated twice with a nuclease cocktail, consisting of DNase 1 (0.005 mg/ml) and RNase A/T1 (0.01 mg/ml) mixture to degrade contaminating nucleic acids and followed by two treatments with proteinase K (0.05 mg/ml) to facilitate removal of contaminating proteins. The resultant solution was concentrated, using a 30,000 molecular mass cutoff membrane by centrifugation, and then underwent two ethanol precipitations; the precipitates were subsequently dried on a bench at room temperature and then stored at −80°C for further CPS purification.

### CPS Fractionation Using Size Exclusion Chromatography

The CPS was separated from LPS by gel filtration chromatography by a modification of the methods as described by Schifferle et al. [26]. The crude CPS was dissolved in 0.05-M glycine, with 0.5% deoxycholate and 0.001 M EDTA, pH 9.8 (5 mg/ml) and applied to a GE HiPrepSephacryl S-400 HR size exclusion column (GE Healthcare, Chicago, IL), pre-equilibrated in the same buffer, and the material was eluted with the deoxycholate containing buffer, using an Akta Avant System (Cytiva Life Sciences, Marlborough, MA) while monitoring a UV signal at 280, 254, and 214 nm, using Unicorn 7.1.0 software. Column fractions were collected, and a sample of each fraction was further characterized by analytical HPLC size exclusion chromatography with multi-angle light scattering coupled to refractive index detection and absorbance at UV 280 nm as described previously [43–45]. An Agilent 1100 HPLC system was used (Agilent, Santa Clara, CA), equipped with degasser, temperature-controlled autosampler (4°C), column compartment (25°C), and coupled to three TOSOH columns in series: TSKgel Guard PWXL 6.0 mm ID × 4.0-cm long, 12-μm particle; TOSOH TSKgel 6000 PWXL 7.8 mm ID × 30-cm long, a 13-μm particle; and a TSKgel 3000 PWXL 7.8 mm ID × 30-cm long, a 7-μm particle (Tosoh Bioscience, Tokyo, Japan). Sample detection was accomplished, using an Agilent 1100 ultraviolet-visible light (UV-VIS) diode array detector (DAD), in addition to a Wyatt Technology DAWN-HELEOS 18-angle laser light scattering detector (MALS) and Wyatt Technology Optilab T-rEX differential refractive interferometer (RI) (Wyatt Technology Corporation, Santa Barbara, CA). A 40–50 μg sample was injected for analysis, using a mobile phase, consisting of 0.2-μm-filtered 1 × PBS with 5% acetonitrile (pH 7.6), running for 60 min at a 0.5-ml/min flow rate. Agilent Open Lab software was used to control the HPLC, and Wyatt Technology Astra 7 software was used for data collection, molecular weight, and protein-conjugate analysis. For the Astra 7 MW analysis, a UV extinction coefficient of 0.9 ml/(mg^*^cm) and a refractive index increment (dn/dc) value of 0.185 ml/g was used for eCRM®, while a dn/dc value of 0.133 and 0.155 ml/g was used for the native *P. gingivalis* CPS and Pg-CV conjugate, respectively, thus enabling a quick determination of the average molecular weight analysis of each eluting fraction. Every other fraction was analyzed *via* SEC-MALS to detect the presence of material with high mass and a minimally associated UV signal, which corresponded to the earliest eluting fractions, and, as such, this was pooled as the CPS product. This result reflects similar observations in previously published CPS purification, where it has been shown that the deoxycholate disrupts the LPS particles, shifting elution of this major contaminant later, the chromatography, thereby permitting isolation of the larger early-eluting CPS component [20, 41]. The final pooled sample was scanned at A_260_ and A_280_ to monitor nucleic acid and protein contamination.

### Polysaccharide Size Reduction With High-Pressure Homogenizer

As purified, the molecular weight of the CPS sample (>10 MDa) was too large for a conjugation; it was subjected to high-pressure mechanical homogenization in order to reduce the overall molecular weight of the polysaccharide in order to facilitate the conjugation to the eCRM® carrier protein. A PandaPlus 2000 homogenizer (GEA, Dusseldorf, Germany) was used to perform multiple passes of the CPS sample (>10,000 psi) to reduce the overall Mw to ~270 kDa as monitored by SEC-MALS in near real time. This homogenized sample was concentrated and washed, using a 30,000 molecular weight cutoff membrane. The sample was subject to purity analysis by SDS-PAGE (data not shown), size/structure analysis *via* SEC-MALS and carbohydrate composition analysis, using high-performance anion-exchange chromatography with pulsed ampherometric detection (HPAEC-PAD) [46].

### Monosaccharide Composition Analysis Based on High-Performance Anion-Exchange Chromatography With Pulsed Amperometric Detection (HPAEC-PAD)

Determination of monosaccharide composition present in the purified CPS was performed, using a Thermo ScientificTMDionex™ ICS-6000 Ion Chromatography system, equipped with a gradient pump, an electrochemical detector with a gold electrode and an Ag/AgCl reference electrode (Thermofisher Scientific, Waltham, MA). A Thermo SceintificTMDionex™ CarboPac PA20 3 × 150 mm analytical column with a guard column was used for separation. Three elution buffers were used, which included 0.45-μm filtered, deionized water with a 18.5-MΩ-cm resistivity, 100-mM NaOH and 100-mM NaOH with 1-M NaOAc, running a custom gradient at 0.5-ml/min for a 30-min collection time. The CPS sample was hydrolyzed using 10-N trifluoro acidic acid (TFA) and heated at 121°C for 2 h to ensure hydrolysis of the polysaccharide to individual monosaccharides. The hydrolyzed sample was then cooled on ice, lyophilized, and suspended in the same initial volume of 0.45-μm filtered, deionized water with an 18.5-MΩ-cm resistivity. The reconstituted sample was subsequently injected onto the HPAEC-PAD system for analysis. Monosaccharide peak identity was verified *via* known retention times of a standard mixture of known monosaccharide composition. The reference mixture used for this study included glycerol phosphate (GroP), glycerol (Gro), rhamnose (Rha), glucosamine (GlcN), galactose (Gal), and glucose (Glc).

### CDAP Activation of Size-Reduced P. gingivalis CPS and Attachment of DBCO-PEG4 Linker

The size-reduced CPS was then activated, using 1-cyano-4-dimethylaminopyridinium tetra fluoroborate (CDAP) [47, 48] in preparation for a conjugation, using copper-free click chemistry to covalently link the polysaccharide *via* a PEG linker to the azide-modified (pAMF) eCRM® carrier protein. In brief, a solution of 2-mM *P. gingivalis* CPS and 100-mM borate was prepared for three molar equivalents of CDAP in acetonitrile with vigorous stirring. After 5 min, 0.5 molar equivalents of dibenzocyclooctyne-amine PEG4 linker (DBCO) were added, and, after incubation at room temperature for 1 h, glycine (pH 8.35) was added to a final concentration of 200 mM to quench the unreacted cyanate esters. The quenched mixture is then incubated for an additional hour; after which, the DBCO-modified polysaccharide was passed through a desalting spin column to remove the remaining reactants.

The purified DBCO-modified *P. gingivalis* CPS was analyzed, using a reverse phase HPLC method to monitor the DBCO concentration at 309 nm during the reaction, as well as to ensure complete removal of unreacted DBCO by the desalting column post reaction (data not shown). This method is used to determine that any residual DBCO-PEG4-Amine linker and unreacted DMAP were completely removed. The polysaccharide concentration was measured, using an anthrone assay [49, 50], and DBCO concentration was measured, using A_309_ nm. These two values are combined to give an estimate of the percentage of modified DBCO-polysaccharide.

### Conjugation of PEG4-Linked *P. gingivalis* Polysaccharide to eCRM®

The eCRM® carrier protein that was used is a modified version of the detoxified diphtheria CRM197 carrier protein engineered to contain the non-native amino acid para-azidomethyl-L-phenylalanine (pAMF) at specific sites within the molecule. The PEG-linked CPS that was generated from the mechanically sized CPS is then conjugated in a site-specific manner to eCRM®. PEG4-DBCO-CPS is in a potassium phosphate buffer, and sodium chloride is mixed well into a tube. An aliquot of eCRM® is added to the CPS mix, and the conjugation reaction is incubated at room temperature overnight. SEC-MALS MW analysis was used to determine the weight-average molar mass for the conjugate and application of the “conjugate-analysis method,” using the differential signal from the ultraviolet signal (a protein-only mass component) and the refractive index signal (a total mass component) to quantify the relative amounts of eCRM® protein and CPS present and percent composition in the final conjugate [43]. This material was used to generate the ultimate vaccine dose administered to mice for the bone-loss study. Samples of the full-length *P. gingivalis* CPS, the mechanically size-reduced polysaccharide, the PEG4/DBCO derivative polysaccharide, and the final polysaccharide/eCRM® conjugate were submitted for an ELISA assay for a positive ID.

### Endotoxin Assessment

The *Limulus* amebocyte lysate (LAL) assay (Endosafe, Charleston, S.C.) was used to assess endotoxin levels at various points throughout the purification and conjugation process, with focus on the final vaccine formulation.

### Oral Bone Loss Modeling

To define oral bone loss in response to *P. gingivalis* and the impact of vaccination, we used the murine oral bone loss model of Baker as we have done previously [20, 21, 51]. Studies using live animals were conducted in accordance with U. Florida IACUC-approved protocols. In brief, Balb-C mice (6 weeks of age; Jackson Labs) were randomly separated into four groups (*n* = 10/group), consisting of an untreated control group, a group only orally challenged with *P. gingivalis*, a group vaccinated with Pg-CV and orally challenged with *P. gingivalis*, and a control vaccination group (CPS only), and orally challenged with *P. gingivalis* (see Figure 2A for an oral bone loss experimental timeline). Following separation, the groups receiving vaccine preparations were initially injected intramuscularly with 50 μl of Pg-CV (7.5-μg delivered injection) or purified CPS (7.5-μg delivered injection), emulsified with Imject (Sigma). Two weeks after the initial vaccinations, the first vaccine boost (50 μl delivering 7.5-μg antigen) was given IM, and 2 weeks following a second boost (50 μl delivering 7.5-μg antigen) was given IM. The untreated and oral-challenge-only groups were monitored during this period. Two weeks following the completion of the second boost, sera were collected from each animal, and all groups except the no-treatment group were orally challenged three times over the course of 1 week with slurry of *P. gingivalis* prepared in pyrogen-free saline with 2% carboxymethylcellulose. Following the oral challenge, mice were rested for 6 weeks; at which time, mice were humanly sacrificed, serum samples were collected, and heads were collected and processed to remove soft tissue. Oral bone loss was determined by morphometric analysis, as previously described [18, 21]. Maxillae were stained with 1% methylene blue, positioned with the buccal aspect infield based on tooth landmarks, and digital images were obtained by a stereo microscope (Leica Microsystems, Germany). The distances from alveolar bone crest (ABC) to the cement-enamel junction (CEJ) were measured on screen, using Leica Application Suite Software, Version 4.12.0 (Leica Microsystems, Germany) from seven sites per hemimaxilla (14 sites/animal). These values were combined with measurements obtained from each animal in the group to achieve a group level mean length, and a standard error of the mean was determined, using PRISM (V8; GraphPad Software, San Diego, CA).

### ELISA-Assessment of Serum Levels of *P. gingivalis* Whole Organism-Specific IgG

IgG from mouse groups was assessed by ELISA as previously reported [20]. In brief, serum samples were added either neat or serial 2-fold diluted to 2% BSA-blocked 96-well-plates, coated with formaldehyde-fixed *P. gingivalis* A7436. Following 2-h incubation at room temperature, wells were washed with PBS-Tween, patted dry, and 1:1,000 dilution of anti-mouse IgG with alkaline phosphatase conjugate (Sigma-Aldrich, USA) was applied to each well for 2 h. Following a second washing step, pNPP substrate (Bio-Rad, USA) was added to each well, color development was halted by the addition of NaOH, and absorbance was read at 405 nm on a Synergy H1 plate reader (Biotek, USA). EC50 values for *P. gingivalis* whole organism-specific IgG were calculated, using absorbance and dilution values as previously reported [21].

### Statistical Analysis

Data were cataloged in PRISM statistical analysis software where subsequent descriptive and comparative analyses were performed. Either *t*-test or ANOVA analysis was performed as described, and a *P* < 0.05 was considered the threshold of significance.

## Results

### SEC-MALS Analysis for Molecular Weight

**Figure 1:**
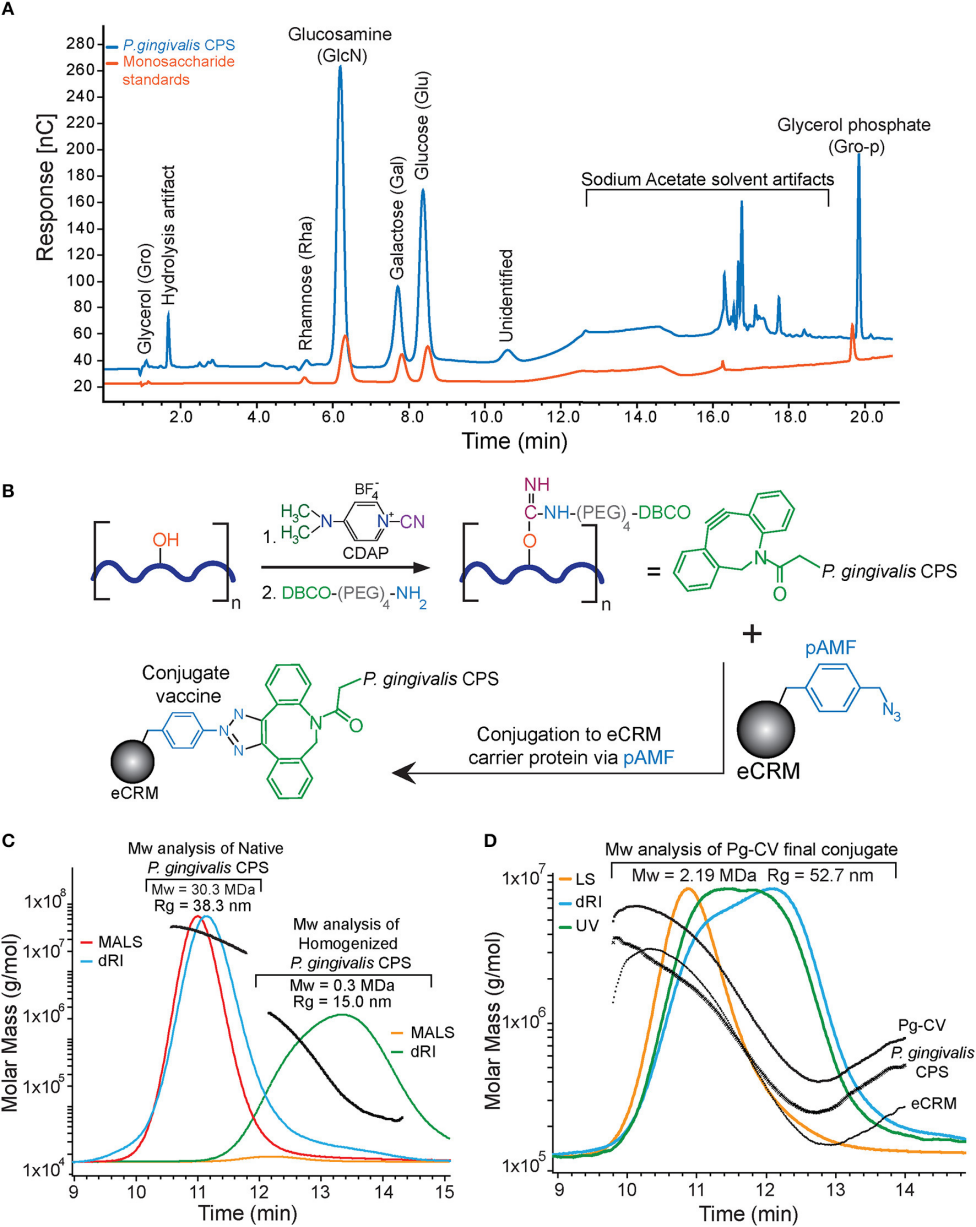
*Porphyromonas gingivalis* A7436 CPS characterization, a conjugation scheme, and analysis of Pg-CV. **(A)** HPAEC-PAD monosaccharide analysis. The Pg-CPS (blue trace) and the monosaccharide reference mixture (orange trace) are presented. Major peaks are labeled with the monosaccharide identity based on retention time and referenced against the reference mixture. **(B)** The schematic conjugation process. The Pg-CPS (dark blue heavy line) with the reactive hydroxyls (red) on the monosaccharides in the repeat unit. Hydroxyl groups were activated with CDAP, resulting in the formation of a cyanoester reactive intermediate. This cyanoester reacts with the terminal amine of the DBCO-PEG4-NH2 linker, forming an amide bond to the polysaccharide *via* the DBCO moiety (green). eCRM® (gray sphere) drawn with one representative pAMF site reacts with the DBCO group, forming the final covalently linked polysaccharide-protein vaccine product: Pg-CV. **(C)** HPLC/SEC-MALS-RI chromatograms of the full-size and size-reduced Pg-CPS. A light scattering signal is a colored red and the refractive index in blue. The later-eluting orange and green peak traces centered between 12 and 14 min represent the light scattering and refractive index signals, respectively. Calculated MW for each eluting time fraction of the chromatograms is overlaid onto the peaks in black. **(D)** HPLC/SEC-MALS-RI-UV chromatograms for the final Pg-CV product. The light scattering signal (orange trace), refractive index (blue trace), and the 280-nm UV signal (green trace) are depicted. Average MW of the final Pg-CPS/eCRM® conjugate product is listed above the peaks and was determined to be 2.19 MDa for final Pg-CV conjugate. The MW results of the protein conjugate analysis are overlaid (black traces) and labeled accordingly.

Molecular weight analysis, using size exclusion chromatography, coupled to light scattering with refractive index and ultraviolet detection, resulted in the ability to monitor the various stages of the vaccine production process, stating with the native full-length CPS, to the mechanically sized polysaccharide, and, finally, the ultimate polysaccharide protein conjugate. MW measurements are summarized in Table 1. The calculated MW for the native full-length *P. gingivalis* CPS was 30.3 MDa and subsequently needed to be processed into short lengths, as, from prior experience, a polysaccharide this large will not undergo an efficient conjugation to the eCRM® carrier, a monomeric protein with a molar mass of around 58.4 kDa. The application of high-pressure homogenization achieved the target MW for a conjugation of <500 kDa; in this case, the final weight average molar mass of the *P. gingivalis* CPS was 270 kDa. This size-reduced *P. gingivalis* CPS was then conjugated to eCRM® (58.4 kDa), forming the final polysaccharide-protein conjugate, Pg-CV, with a weight average molar mass between 2.2 and 2.7 MDa. The final vaccine conjugate drug product was formulated at a concentration of 0.95 mg/ml in PBS pH 7.4 and stored at −80°C. The final Pg-CV product was 0.49 mg/m *P. gingivalis* CPS (Figure 1D) or ~48% capsular polysaccharide by weight.

**Table 1:** SEC-MALS Mwt summary.

**Material**	**Mw (kDa)**	**% protein**	**% polysaccharide**
Native PGPS	30,315	n/a	n/a
Sized PGPS	270	n/a	n/a
Sized PGPS eCRM Conjugate	2,185	51.2	48.7
dn/dc PS = 0.133 mL/g; dn/dc Protein = 0.185 mL/g;			
Ext.Coeff.eCRM = 0.9 mL/(mg cm); Ext.Coeff.PS = 0.0 mL/(mg cm)			

*Molecular weight measurements of the P. gingivalis CPS at the various stages in the production of the final vaccine. The relative composition of polysaccharide and protein in the final conjugate product is based on the differential detector responses in the dRI and UV, as described in the text*.

### Endotoxin Assessment

All purified CPS fractions and final vaccine formulations were found to be free of any contaminating endotoxin/LPS (<100 EU/ml, data not shown).

### HPAEC-PAD Analysis for Capsule Monosaccharide Composition

The HPAE-PAD monosaccharide analysis revealed compositional similarity to the purified *P. gingivalis* CPS polysaccharide composition with prior studies [26], and yet differences are present as would be expected between CPS from different *P. gingivalis* strains. A representative HPAEC-PAD chromatogram of the purified *P. gingivalis* CPS, as well as the monosaccharide standard mixture, is shown in Figure 1A. Our initial analysis for the isolated *P. gingivalis* CPS suggests the presence of four main monosaccharide peaks: glucosamine, galactose, glucose, and glycerol phosphate, at a molar ratio of 5GlcN: 1Gal: 3Glc: 1GroP (i.e., 1 molar equivalent per PSRU). In addition, some minor peaks were observed with a potential rhamnose peak (retention time around 4.25 min) and a yet-to-be-identified peak at 10.5 min. The remaining peaks are speculated to be either hydrolysis artifacts or peaks associated with the background buffer signals (12–19 min). The lack of mannose (Man) and N-acetylgalactosamine (GalNac) signatures supports that the purified *P. gingivalis* CPS was free of the two major contaminating lipopolysaccharides (LPS) synthesized by the bacteria, mainly O-LPS, which has O-antigen tetrasaccharide repeat units and A-LPS, which has anionic polysaccharide repeating units [52, 53]. Interestingly, the monosaccharide composition determined for *P. gingivalis* CPS is similar to several pneumococcal polysaccharides such as the *S. pneumonia* Strain 15B, which has PSRU composed of 1GlcNAc: 3Gal: 1Glc: 1Gro [54]. Without any prior knowledge of activation and reaction conditions for this CPS, the similarity between sugar composition to *S. pneumonia* serotype 15B suggested that the activation and conjugation conditions used for 15B could be applied to *P. gingivalis* CPS and Vaxcyte eCRM® [36] (Figures 1B,C).

### Pg-CV Conjugate Vaccine Elicits a Robust IgG Response From Mice

**Figure 2:**
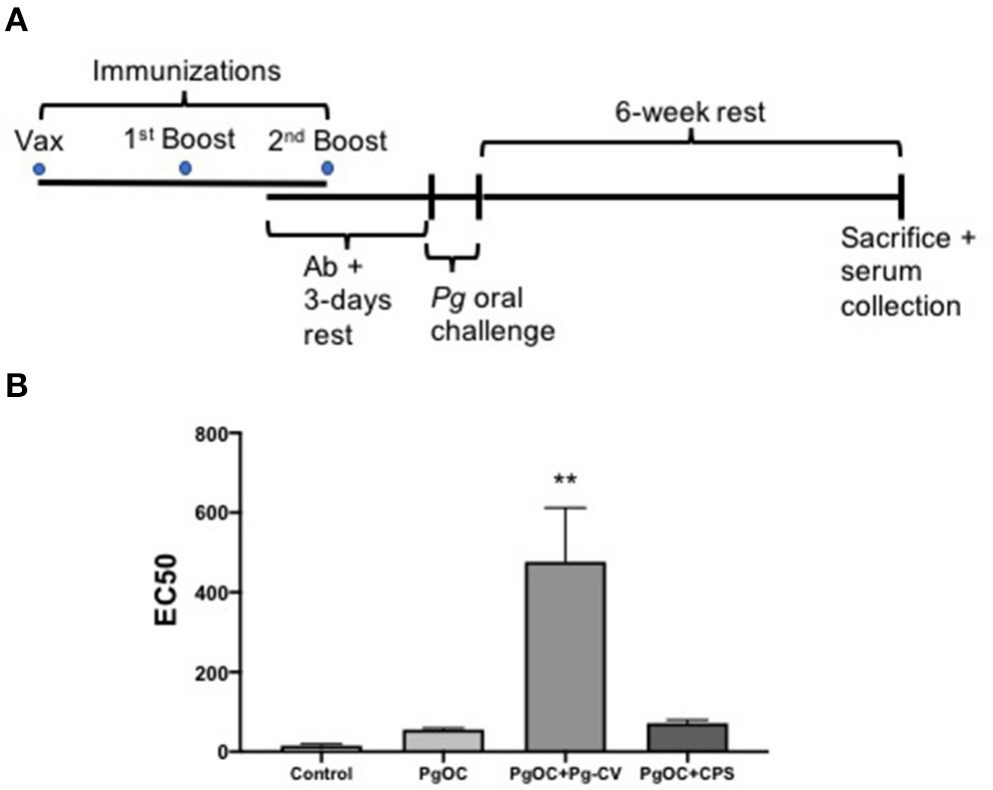
Pg-CV elicits a potent *P. gingivalis* whole organism-specific IgG host response. **(A)** A timeline of animal studies. Groups of animals (*n* = 10/group) did not receive injection or were immunized with Pg-CV or purified *P. gingivalis* CPS by intramuscular immunization three times in a 4-week period. Following oral antibiotic administration (Ab), animals were challenged orally three times in a 1-week period with *P. gingivalis* strain A7436 in 2% carboxymethylcellulose. Mice were humanely sacrificed 6 weeks later, sera were collected, and oral bone loss was assessed, using digital microscopy; **(B)** EC50 values of groups at sacrifice were determined based on initial dilution of harvested serum from pooled group samples (2–3 samples/pool, *n* = 4 pools/group); **P < 0.01 vs. Control, PgOC, and PgOC + CPS by ANOVA, using Tukey post-test (no other comparisons were significant).

A conjugation of relevant capsule polysaccharides has been shown to enhance the vaccine efficacy of these molecules over pure capsular polysaccharides. Previously, we reported that, in pure form, the *P. gingivalis* CPS was able to provide a measure of protection to mice orally challenged with this organism but did not elicit a robust IgG response as compared with a heat-killed whole organism [20]. To understand the impact of Pg-CV as a vaccine for periodontal disease, we used a murine oral challenge model. Circulating levels of *P. gingivalis*, whole organism-specific IgG was determined in serum collected from each animal by an enzyme-linked immunosorbent assay. At grouping, all animals possessed very low levels of *P. gingivalis*-specific IgG (data not shown). Serum samples collected from groups after completion of the three rounds of vaccination, but, immediately before oral challenge, expectedly showed that the non-vaccinated control group and the group scheduled for *P. gingivalis* oral challenge remained essentially negative for *P. gingivalis* IgG (Supplemental Figure 1). The group of mice immunized with Pg-CV developed a significantly enhanced IgG response that robustly detected whole *P. gingivalis* when compared with non-vaccinated mice, while the group immunized with the purified CPS alone developed only a slight increase in *P. gingivalis*-specific IgG (Supplemental Figure 1). At sacrifice control, animals remained essentially negative, while the group orally challenged with *P. gingivalis* developed only a modest increase in IgG response as compared with the no immunization/no oral challenge control group. Animals receiving Pg-CV continued to express significantly high levels of IgG to this organism as compared with the other groups (*P* < 0.05 for all; Figure 2B), while the group of mice receiving *P. gingivalis* CPS developed only a modest IgG response to a whole organism.

### Vaccination With the Conjugate Vaccine Limits *P. gingivalis*-Elicited Oral Bone Loss

**Figure 3:**
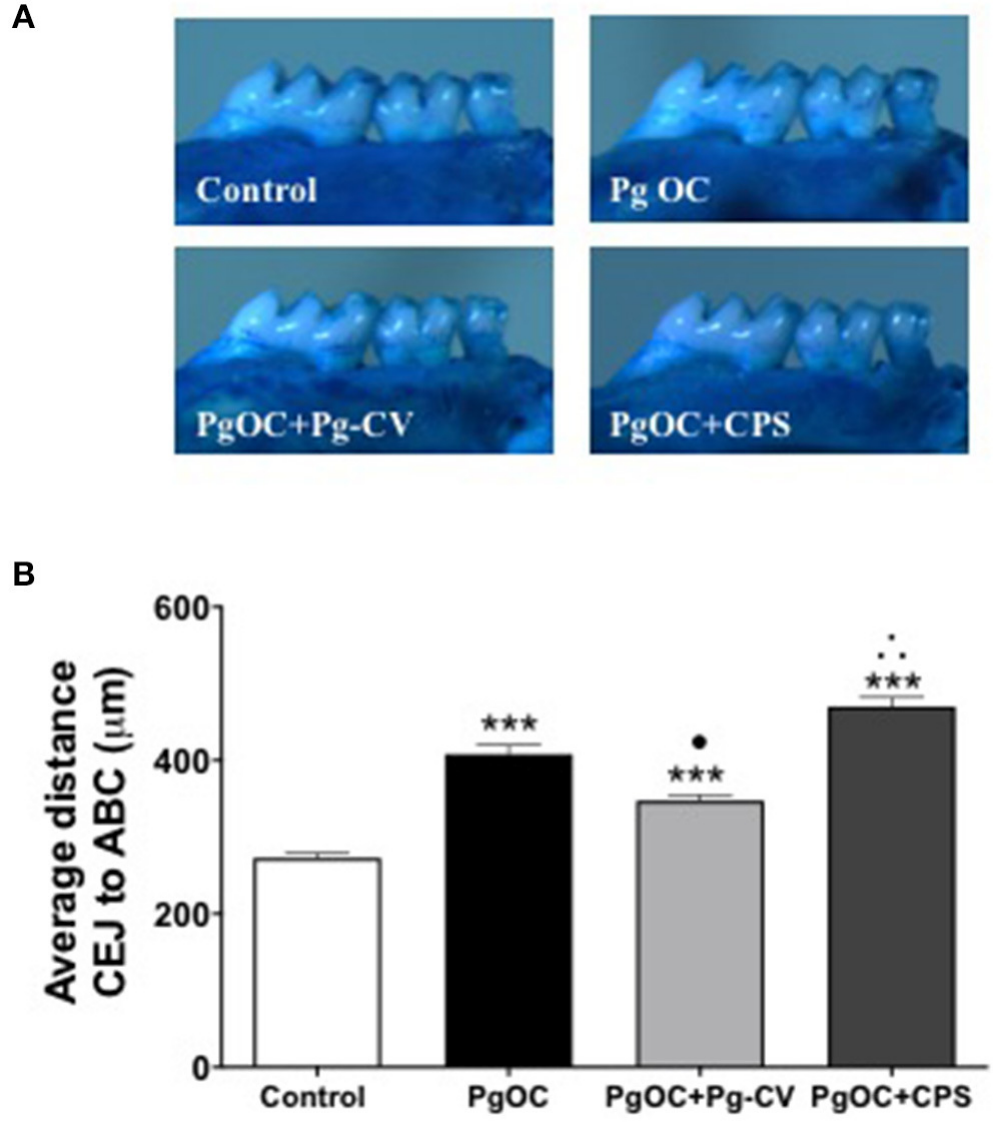
Pg-CV vaccination protects animals from subsequent oral infection-elicited bone loss. **(A)** Representative images of an untreated group (Control), *P. gingivalis* oral challenge (Pg OC), *P. gingivalis* oral challenge + immunized with Pg-CV (PgOC + Pg-CV) and *P. gingivalis* oral challenge + immunized with *P. gingivalis* CPS + (PgOC + CPS). **(B)** Oral bone levels from experimental groups. Mean distance from the alveolar bone crest (ABC) to cementum enamel junction (CEJ) was measured at 14 landmark sites (7 on each hemimaxilla) per animal (*n* = 10 mice/group), and measurement data were pooled to achieve group-level mean and SEM. ***P < 0.001 compared with Control, •*P* < 0.05 vs. Pg OC (decrease), ∴*P* < 0.05 vs. Pg OC (increase) by ANOVA with Tukey post-test.

The mouse model has been used successfully by our group and others to assess various vaccine formulations and their preclinical efficacy in preventing oral bone loss [20, 21]. To understand the effect of Pg-CV on *P. gingivalis* oral bone loss, after sacrifice, the harvested, cleaned, and stained maxillae were measured to define oral bone loss levels for each group (Figure 3A). ABC to CEJ measurements in the control group established the typical established bone levels for the groups. As expected, *P. gingivalis* oral challenge elicited a robust oral bone loss pattern (*P* < 0.05 vs. control; Figure 3B). Immunization with Pg-CV significantly limited oral bone loss in the group of mice orally challenged with *P. gingivalis*. Unexpectedly, we observed a slight increase in the ABC-to-CEJ distance in the group immunized with the CPS alone as compared with the *P. gingivalis* oral challenge group (*P* < 0.05; Figure 3B).

## Discussion

Conjugate vaccines have proved to be an effective strategy to limit the impact of several significant human diseases, including *S. pneumonia*, and other pneumococci [55]. Periodontal disease is a chronic inflammatory disease that is exceedingly difficult to manage clinically with high expectations of a good long-term prognosis for staving the progressive loss of periodontal destruction and associated tooth loss as lack of effective therapies have continued to evade clinicians and lead to a poor patient prognosis. As such, the development of novel approaches to prevent or limit the progression of periodontal disease is highly attractive and would change the impact of this disease in humans. An array of *P. gingivalis* molecules have been tested in an expanding number of studies investigating suitability for vaccine potential. Although results are mixed, several molecules have been shown to provide meaningful protection from infection-elicited oral bone loss, including the CPS of *P. gingivalis* [20, 29].

Early studies established that encapsulated *P. gingivalis* strains caused more aggressive infections when injected into mice [22]. Specifically, strains that produce a capsule cause a spreading type of infection, with *P. gingivalis* recovery from blood, spleen, and kidneys, while non-encapsulated strains (381 and 33,277) cause localized abscesses, suggesting that encapsulation is linked to dissemination. In addition, Aduse-Opoku et al. [25] identified the capsule biosynthetic locus, and Davey and Duncan [56] showed that capsule production blocked surface attachment and biofilm development. Interestingly, cloning of the CPS locus (PG0106-PG0120 and a promoter region just upstream of PG0106) into plasmid PT-COW and introducing this plasmid (PT-Capsule) into a non-encapsulated *P. gingivalis* strain 381Δ0106 mutant resulted in capsule expression and inhibition of biofilm formation, showing that production of capsule interferes with the adhesion properties of fimbriae (Supplemental Figure 2). Thus, a capsule plays a dominant role in defining the surface properties of this organism. These studies began to pave the way for a more detailed understanding of the contribution of a *P. gingivalis* capsule to the infection potential of this organism. Although it is understood that *P. gingivalis* strains are typable based on serologic differences in the capsules of this organism ([23, 57], and [58]), the consensus of capsule structure is lacking as existing reports provide a mixed picture of the capsule repeat structure [26, 27]. Importantly, work by Choi et al. [59] showed that, in SCID mice reconstituted with human peripheral blood leukocytes, the use of *P. gingivalis* CPS as part of a conjugate vaccine possessing fimbrial proteins purified from this organism induced a robust human IgG response in the sera of these animals and protected immunized animals from subsequent subcutaneous *P. gingivalis* challenge. More recently, Gonzalez et al. [20] have reported that injection of mice with purified CPS of *P. gingivalis* protected animals from subsequent oral bone loss; however, production of organism-specific immunoglobulins capable of detecting whole *P. gingivalis* organisms in response to vaccination was modest compared with levels observed in animals that received immunization with a killed whole organism. We observed that Pg-CV elicited a significantly more robust *P. gingivalis*-specific IgG response than what was observed in the group that provided the polysaccharide alone.

The purified *P. gingivalis* CPS was too large to efficiently conjugate when first purified (~30.3 MDa); it was, therefore, necessary to reduce the overall molar mass size of the CPS. Surprisingly the *P. gingivalis* CPS required an extended homogenization time, using very high sheer pressure (20,000 PSI). Also of note is that the initial attempts to accomplish size reduction, using acetic acid hydrolysis, were not very effective (data not shown). Taken together, these characteristics align with a presumed biological function of the capsule to protect from the environment, in particular the pH-shielding function of the capsular polysaccharide *in vivo*. Additional characterizations of the *P. gingivalis* CPS, using LS-MS, as well as NMR experiments, are currently in progress in order to fully characterize the linkage and structure of this capsular polysaccharide.

Generally speaking, it is understood that most bacterial capsules are T cell-independent antigens, and that, following injection, the host develops only a low-level IgG response. Certain conjugate vaccines have been found to possess a greater demonstrated capacity to elicit specific antibody responses [60, 61]. Using our conjugation technology, these findings support that the conjugated form of *P. gingivalis* CPS was a more potent inducer of whole organism-specific IgG than what was found with CPS alone. In these initial studies, we focused our attention on the endpoints of IgG production and protection from *P. gingivalis*-elicited oral bone loss. Although one animal died during our experiments, that death occurred in the group that had only received *P. gingivalis* oral challenge and thus did not represent the associated toxicity of Pg-CV to the host. An unexpected finding in the present study was that the use of CPS alone as an immunogen did not provide a measure of protection; rather, it appeared to potentially exacerbate the loss of oral bone levels. Previously, Gonzalez et al. [20] reported that immunization with purified *P. gingivalis* CPS alone protected mice from subsequent live *P. gingivalis* oral challenge-elicited oral bone loss. We do not fully understand the different findings between our studies and those performed previously in regard to the unconjugated capsule and its capacity to protect from infection-elicited oral bone loss. Although several factors are similar between these studies, including the overall immunization period and the mouse strain employed, there were several significant differences in experimentation, including a reduced number of injections to immunize mice in the present study (1/2 than previously reported); >10-fold less CPS was delivered per injection to recipient animals in the present study (100 μg/injection CPS previously vs. 7.5 μg/injection in the present study), and differences in the route of immunization (intramuscular in the present study vs. subcutaneous in the prior study). One concept that may explain the differences observed between the two studies may be related to vaccine-associated enhanced disease [62, 63]. As we do not understand the mechanisms of action of our vaccine, we are not currently in a position to fully explain why differences in vaccine approaches lead to specific quantifiable differences in outcomes between our two studies. Thus, further investigations are needed to optimize the amount of an antigen delivered to recipients as well as a vaccine schedule to maximize vaccine effectiveness. Importantly, however, the present study shows that, when conjugated to eCRM®, the CPS of *P. gingivalis* protected animals from oral challenge-elicited oral bone loss.

We chose *P. gingivalis* strain A7436 as it is a representative capsule serotype K1 strain. Previous work has shown that a capsule expressing strains causes more significant infections when tested *in vivo* than what observed with K-strains [22]. Importantly, K1 *P. gingivalis* has been reported more prevalent than other K serotypes in periodontitis patients [64], although other K strain serotypes are also associated with disease [23]. These findings support our focus on *P. gingivalis* K-antigen as a tenable target for vaccine development and further support our initial targeting of K1 serotypes, and, indeed, in our hands, immunization of mice with the K1-based Pg-CV prior to homologous *P. gingivalis* oral challenge was found to provide a measure of protection from oral live *P. gingivalis* challenge. These findings provide proof of a concept that our Pg-CV preparation may be effective for periodontal disease treatment. One limitation of our study is that we currently focus on the K1 capsule. We envision that the development of an optimal *P. gingivalis* capsular polysaccharide-based vaccine would require the use of several K antigens as part of a conjugate vaccine to cover the majority of K serotypes that associate with periodontal disease in humans and will be a vaccine optimization focus of future studies.

In summary, our findings support that our Pg-CV, when injected in a preclinical model of oral bone loss, in a manner consistent with human vaccination paradigms, including an intramuscular route and alum adjuvant, effectively elicits a robust IgG response that recognizes *P. gingivalis* organisms. Most importantly, this vaccine candidate demonstrates characteristics to support; it may represent a novel and potentially effective tool to prevent the extensive oral bone loss that is a characteristic of human periodontal disease.

## Data Availability Statement

The datasets generated for this article are not readily available because It would be requested that the recipient sign a non-disclosure agreement to Vaxcyte, before release of the data to them. Requests to access the datasets should be directed to Jeff Fairman, jeff.fairman@vaxcyte.com.

## Ethics Statement

The animal study was reviewed and approved by University of Florida (UF) Institutional Animal Care and Use Committee (IACUC).

## Author Contributions

JF, MD, and FG conceptualized and designed the experiments, evaluated and analyzed data, and contributed to the writing of the manuscript. FR grew bacteria, performed mouse experiments, collected samples, performed ELISAs, collected data, performed data analysis, and contributed to manuscript writing. SG performed biofilm assays and contributed to manuscript writing. AB and AS performed capsular polysaccharide isolation, purification, purity assessments, capsule conjugations, and contributed to the manuscript writing. NK and LP produced the carrier protein. NK assisted with manuscript figures. All authors contributed to the article and approved the submitted version.

## Conflict of Interest

AB, AS, NK, LP, and JF are current employees of Vaxcyte Inc.; JF is a founding member of Vaxcyte. The remaining authors declare that the research was conducted in the absence of any commercial or financial relationships that could be construed as a potential conflict of interest.

## Abbreviations

ABC: alveolar bone crest
AKTA: genuine, authentic, pure, undiluted
ANOVA: analysis of variance
A-LPS: lipopolysaccharide with anionic polysaccharide repeating units
CDAP: 1-cyano-4-dimethylaminopyridinium tetra fluoroborate
CEJ: cementum-enamel junction
CPS: capsular polysaccharide; in this manuscript, specifically from *P. gingivalis*
CRM197: non-toxic mutant of diphtheria toxin, used as a carrier protein
CV: conjugate vaccine
DBCO: Dibenzocyclooctyne-amine
DMAP: 4-Dimethylaminopyrimidine
dRI: differential refractive index; used interchangeably with RI
eCRM®: proprietary version of CRM197 carrier protein developed by Vaxcyte, Inc.
EDTA: Ethylenediaminetetraacetic acid
ELISA: enzyme linked immunosorbent assay
Gal: galactose
GalNAc: N-acetylgalactosamine
Glu: glucose
GluN: glucosamine
Gro: glycerol
Gro-P: glycerol phosphate
Hib: *Haemophilus influenza* type b
HPAEC-PAD: high performance anion exchange chromatography with pulsed ampherometric detection
HPLC: high-performance liquid chromatography
IACUC: Institutional Animal Care and Use Committee
ID: identity
IgG: immunoglobulin G
IM: intramuscular
LAL: limulus ameobocyte lysate assay
LPS: lipopolysaccharide endotoxin
MALS: multi-angle light scattering
Man: mannose
MW: molar mass
nnAAs: non-native amino acids
UV: ultraviolet
Rha: rhamnose
RI: refractive index; used interchangeably with dRI
SEC: size-exclusion chromatography
SCID: severe combined immune deficient
SDS: sodium dodecyl sulfate
OC: oral challenge
O-LPS: lipopolysaccharide with O-antigen tetra-saccharide repeat units
PAGE: polyacrylamide gel electrophoresis
pAMF: para-azidomethyl-L-phenylalanine
PBS: phosphate buffered saline
PEG: polyethylene glycol linker molecule
Pg: *Porphyromonas gingivalis*
Pg-CV: *Porphyromonas gingivalis* capsular polysaccharide + eCRM® conjugate vaccine
Pg-CPS: *Porphyromonas gingivalis* capsular polysaccharide
PSRU: polysaccharide repeat unit.

## References

[1] Righolt AJ Jevdjevic M Marcenes W Listl S. Global-, regional-, and country-level economic impacts of dental diseases in 2015. J Dent Res. (2018) 97:501–7. 10.1177/0022034517750572 29342371

[2] Eke PI Thornton-Evans GO Wei L Borgnakke WS Dye BA Genco RJ. Periodontitis in US Adults: National Health and Nutrition Examination Survey 2009-2014. J Am Dent Assoc. (2018) 149:576–88 e6. 10.1016/j.adaj.2018.04.023 29957185 PMC8094373

[3] Listl S Galloway J Mossey PA Marcenes W. Global economic impact of dental diseases. J Dent Res. (2015) 94:1355–61. 10.1177/0022034515602879 26318590

[4] Cahill TJ Harrison JL Jewell P Onakpoya I Chambers JB Dayer M. Antibiotic prophylaxis for infective endocarditis: a systematic review and meta-analysis. Heart. (2017) 103:937–44. 10.1136/heartjnl-2015-309102 28213367

[5] Kriebel K Hieke C Muller-Hilke B Nakata M Kreikemeyer B. Oral biofilms from symbiotic to pathogenic interactions and associated disease -connection of periodontitis and rheumatic arthritis by peptidylarginine deiminase. Front Microbiol. (2018) 9:53. 10.3389/fmicb.2018.00053 29441048 PMC5797574

[6] Olsen I Yilmaz O. Possible role of *Porphyromonas gingivalis* in orodigestive cancers. J Oral Microbiol. (2019) 11:1563410. 10.1080/20002297.2018.1563410 30671195 PMC6327928

[7] Dominy SS Lynch C Ermini F Benedyk M Marczyk A Konradi A. *Porphyromonas gingivalis* in Alzheimer's disease brains: evidence for disease causation and treatment with small-molecule inhibitors. Sci Adv. (2019) 5:eaau3333. 10.1126/sciadv.aau3333 30746447 PMC6357742

[8] Griffen AL Beall CJ Campbell JH Firestone ND Kumar PS Yang ZK. Distinct and complex bacterial profiles in human periodontitis and health revealed by 16S pyrosequencing. ISME J. (2012) 6:1176–85. 10.1038/ismej.2011.191 22170420 PMC3358035

[9] Singh A Wyant T Anaya-Bergman C Aduse-Opoku J Brunner J Laine ML. The capsule of *Porphyromonas gingivalis* leads to a reduction in the host inflammatory response, evasion of phagocytosis, and increase in virulence. Infect Immun. (2011) 79:4533–42. 10.1128/IAI.05016-11 21911459 PMC3257911

[10] Herath TDK Darveau RP Seneviratne CJ Wang CY Wang Y Jin L. Heterogeneous *Porphyromonas gingivalis* LPS modulates immuno-inflammatory response, antioxidant defense and cytoskeletal dynamics in human gingival fibroblasts. Sci Rep. (2016) 6:29829. 10.1038/srep29829 27538450 PMC4990928

[11] Potempa J Sroka A Imamura T Travis J. Gingipains, the major cysteine proteinases and virulence factors of *Porphyromonas gingivalis*: structure, function and assembly of multidomain protein complexes. Curr Protein Pept Sci. (2003) 4:397–407. 10.2174/1389203033487036 14683426

[12] Aruni AW Robles A Fletcher HM. VimA mediates multiple functions that control virulence in *Porphyromonas gingivalis*. Mol Oral Microbiol. (2013) 28:167–80. 10.1111/omi.12017 23279905 PMC3625487

[13] O'Brien-Simpson NM Holden JA Lenzo JC Tan Y Brammar GC Walsh KA. A therapeutic *Porphyromonas gingivalis* gingipain vaccine induces neutralising IgG1 antibodies that protect against experimental periodontitis. NPJ Vaccines. (2016) 1:16022. 10.1038/npjvaccines.2016.22 29263860 PMC5707886

[14] Yoshino T Laine ML van Winkelhoff AJ Dahlen G. Genotype variation and capsular serotypes of *Porphyromonas gingivalis* from chronic periodontitis and periodontal abscesses. FEMS Microbiol Lett. (2007) 270:75–81. 10.1111/j.1574-6968.2007.00651.x 17439635

[15] Kato T Kawai S Nakano K Inaba H Kuboniwa M Nakagawa I. Virulence of *Porphyromonas gingivalis* is altered by substitution of fimbria gene with different genotype. Cell Microbiol. (2007) 9:753–65. 10.1111/j.1462-5822.2006.00825.x 17081195

[16] Takahashi Y Kumada H Hamada N Haishima Y Ozono S Isaka M. Induction of immune responses and prevention of alveolar bone loss by intranasal administration of mice with *Porphyromonas gingivalis* fimbriae and recombinant cholera toxin B subunit. Oral Microbiol Immunol. (2007) 22:374–80. 10.1111/j.1399-302X.2007.00373.x 17949339

[17] Puth S Hong SH Park MJ Lee HH Lee YS Jeong K. Mucosal immunization with a flagellin-adjuvanted Hgp44 vaccine enhances protective immune responses in a murine *Porphyromonas gingivalis* infection model. Hum Vaccin Immunother. (2017) 13:2794–803. 10.1080/21645515.2017.1327109 28604268 PMC5718812

[18] Gibson FC 3rd Genco CA. Prevention of *Porphyromonas gingivalis*-induced oral bone loss following immunization with gingipain R1. Infect Immun. (2001) 69:7959–63. 10.1128/IAI.69.12.7959-7963.2001 11705986 PMC98900

[19] Miyachi K Ishihara K Kimizuka R Okuda K. Arg-gingipain A DNA vaccine prevents alveolar bone loss in mice. J Dent Res. (2007) 86:446–50. 10.1177/154405910708600511 17452566

[20] Gonzalez D Tzianabos AO Genco CA Gibson FC 3rd. Immunization with *Porphyromonas gingivalis* capsular polysaccharide prevents *P. gingivalis*-elicited oral bone loss in a murine model. Infect Immun. (2003) 71:2283–7. 10.1128/IAI.71.4.2283-2287.2003 12654858 PMC152101

[21] Huang N Shimomura E Yin G Tran C Sato A Steiner A. Immunization with cell-free-generated vaccine protects from *Porphyromonas gingivalis*-induced alveolar bone loss. J Clin Periodontol. (2019) 46:197–205. 10.1111/jcpe.13047 30578564 PMC7891626

[22] Laine ML van Winkelhoff AJ. Virulence of six capsular serotypes of *Porphyromonas gingivalis* in a mouse model. Oral Microbiol Immunol. (1998) 13:322–5. 10.1111/j.1399-302X.1998.tb00714.x 9807125

[23] Laine ML Appelmelk BJ van Winkelhoff AJ. Prevalence and distribution of six capsular serotypes of *Porphyromonas gingivalis* in periodontitis patients. J Dent Res. (1997) 76:1840–4. 10.1177/00220345970760120601 9390477

[24] Bainbridge BW Hirano T Grieshaber N Davey ME. Deletion of a 77-base-pair inverted repeat element alters the synthesis of surface polysaccharides in *Porphyromonas gingivalis*. J Bacteriol. (2015) 197:1208–20. 10.1128/JB.02589-14 25622614 PMC4352660

[25] Aduse-Opoku J Slaney JM Hashim A Gallagher A Gallagher RP Rangarajan M. Identification and characterization of the capsular polysaccharide (K-antigen) locus of *Porphyromonas gingivalis*. Infect Immun. (2006) 74:449–60. 10.1128/IAI.74.1.449-460.2006 16369001 PMC1346596

[26] Schifferle RE Reddy MS Zambon JJ Genco RJ Levine MJ. Characterization of a polysaccharide antigen from *Bacteroides gingivalis*. J Immunol. (1989) 143:3035–42. 2509563

[27] Farquharson SI Germaine GR Gray GR. Isolation and characterization of the cell-surface polysaccharides of *Porphyromonas gingivalis* ATCC 53978. Oral Microbiol Immunol. (2000) 15:151–7. 10.1034/j.1399-302x.2000.150302.x 11154397

[28] d'Empaire G Baer MT Gibson FC III. The K1 serotype capsular polysaccharide of *Porphyromonas gingivalis* elicits chemokine production from murine macrophages that facilitates cell migration. Infect Immun. (2006) 74:6236–43. 10.1128/IAI.00519-06 16940143 PMC1695525

[29] Schifferle RE Chen PB Davern LB Aguirre A Genco RJ Levine MJ. Modification of experimental *Porphyromonas gingivalis* murine infection by immunization with a polysaccharide-protein conjugate. Oral Microbiol Immunol. (1993) 8:266–71. 10.1111/j.1399-302X.1993.tb00572.x 8265201

[30] Sun L Middleton DR Wantuch PL Ozdilek A Avci FY. Carbohydrates as T-cell antigens with implications in health and disease. Glycobiology. (2016) 26:1029–40. 10.1093/glycob/cww062 27236197 PMC6086537

[31] Mitchell R Kelly DF Pollard AJ Truck J. Polysaccharide-specific B cell responses to vaccination in humans. Hum Vaccin Immunother. (2014) 10:1661–8. 10.4161/hv.28350 24632599 PMC5396230

[32] Gold R Lepow ML Goldschneider I Draper TL Gotschlich EC. Clinical evaluation of group A and group C meningococcal polysaccharide vaccines in infants. J Clin Invest. (1975) 56:1536–47. 10.1172/JCI108235 1202084 PMC333132

[33] Pichichero ME. Protein carriers of conjugate vaccines: characteristics, development, and clinical trials. Hum Vaccin Immunother. (2013) 9:2505–23. 10.4161/hv.26109 23955057 PMC4162048

[34] Pace D. Glycoconjugate vaccines. Expert Opin Biol Ther. (2013) 13:11–33. 10.1517/14712598.2012.725718 22992106

[35] Broker M. Potential protective immunogenicity of tetanus toxoid, diphtheria toxoid and Cross Reacting Material 197 (CRM197) when used as carrier proteins in glycoconjugates. Hum Vaccin Immunother. (2016) 12:664–7. 10.1080/21645515.2015.1086048 26327602 PMC4964734

[36] Fairman JC Heinrichs JH Chan W Marcq OJG Behrens CR. Conjugate Vaccines. United States Patent Application No. 20200054739/A1 (2020).

[37] Giannini G Rappuoli R Ratti G. The amino-acid sequence of two non-toxic mutants of diphtheria toxin: CRM45 and CRM197. Nucleic Acids Res. (1984) 12:4063–9. 10.1093/nar/12.10.4063 6427753 PMC318816

[38] Choe S Bennett MJ Fujii G Curmi PM Kantardjieff KA Collier RJ. The crystal structure of diphtheria toxin. Nature. (1992) 357:216–22. 10.1038/357216a0 1589020

[39] Diethelm-Okita BM Okita DK Banaszak L Conti-Fine BM. Universal epitopes for human CD4+ cells on tetanus and diphtheria toxins. J Infect Dis. (2000) 181:1001–9. 10.1086/315324 10720523

[40] Raju R Navaneetham D Okita D Diethelm-Okita B McCormick D Conti-Fine BM. Epitopes for human CD4+ cells on diphtheria toxin: structural features of sequence segments forming epitopes recognized by most subjects. Eur J Immunol. (1995) 25:3207–14. 10.1002/eji.1830251202 8566002

[41] Alvarez CA Cobb BA. Purification of capsular polysaccharide complex from Gram-negative bacteria. Methods Mol Biol. (2019) 1954:25–35. 10.1007/978-1-4939-9154-9_3 30864121

[42] Gibson FC III Tzianabos AO Onderdonk AB. The capsular polysaccharide complex of *Bacteroides fragilis* induces cytokine production from human and murine phagocytic cells. Infect Immun. (1996) 64:1065–9. 10.1128/iai.64.3.1065-1069.1996 8641762 PMC173883

[43] Wen J Arakawa T Philo JS. Size-exclusion chromatography with on-line light-scattering, absorbance, and refractive index detectors for studying proteins and their interactions. Anal Biochem. (1996) 240:155–66. 10.1006/abio.1996.0345 8811899

[44] Kendrick BS Kerwin BA Chang BS Philo JS. Online size-exclusion high-performance liquid chromatography light scattering and differential refractometry methods to determine degree of polymer conjugation to proteins and protein-protein or protein-ligand association states. Anal Biochem. (2001) 299:136–46. 10.1006/abio.2001.5411 11730335

[45] Wyatt P. Light scattering and the absolute characterization of macromolecules. Analytica Chimica Acta. (1993) 272:1–40. 10.1016/0003-2670(93)80373-S25531696

[46] Rohrer JS Basumallick L Hurum D. High-performance anion-exchange chromatography with pulsed amperometric detection for carbohydrate analysis of glycoproteins. Biochemistry. (2013) 78:697–709. 10.1134/S000629791307002X 24010833

[47] Shafer DE Toll B Schuman RF Nelson BL Mond JJ Lees A. Activation of soluble polysaccharides with 1-cyano-4-dimethylaminopyridinium tetrafluoroborate (CDAP) for use in protein-polysaccharide conjugate vaccines and immunological reagents. II. Selective crosslinking of proteins to CDAP-activated polysaccharides. Vaccine. (2000) 18:1273–81. 10.1016/S0264-410X(99)00370-9 10649629

[48] Lees A Nelson BL Mond JJ. Activation of soluble polysaccharides with 1-cyano-4-dimethylaminopyridinium tetrafluoroborate for use in protein-polysaccharide conjugate vaccines and immunological reagents. Vaccine. (1996) 14:190–8. 10.1016/0264-410X(95)00195-7 8920699

[49] Ludwig TG Goldberg JV. The anthrone method for the determination of carbohydrates in foods and in oral rinsing. J Dent Res. (1956) 35:90–4. 10.1177/00220345560350012301 13286391

[50] Laurentin A Edwards CA. A microtiter modification of the anthrone-sulfuric acid colorimetric assay for glucose-based carbohydrates. Anal Biochem. (2003) 315:143–5. 10.1016/S0003-2697(02)00704-2 12672425

[51] Baker PJ Evans RT Roopenian DC. Oral infection with *Porphyromonas gingivalis* and induced alveolar bone loss in immunocompetent and severe combined immunodeficient mice. Arch Oral Biol. (1994) 39:1035–40. 10.1016/0003-9969(94)90055-8 7717884

[52] Paramonov N Bailey D Rangarajan M Hashim A Kelly G Curtis MA. Structural analysis of the polysaccharide from the lipopolysaccharide of *Porphyromonas gingivalis* strain W50. Eur J Biochem. (2001) 268:4698–707. 10.1046/j.1432-1327.2001.02397.x 11532006

[53] Paramonov N Rangarajan M Hashim A Gallagher A Aduse-Opoku J Slaney JM. Structural analysis of a novel anionic polysaccharide from *Porphyromonas gingivalis* strain W50 related to Arg-gingipain glycans. Mol Microbiol. (2005) 58:847–63. 10.1111/j.1365-2958.2005.04871.x 16238632

[54] van Selm S van Cann LM Kolkman MA van der Zeijst BA van Putten JP. Genetic basis for the structural difference between *Streptococcus pneumoniae* serotype 15B and 15C capsular polysaccharides. Infect Immun. (2003) 71:6192–8. 10.1128/IAI.71.11.6192-6198.2003 14573636 PMC219561

[55] Berical AC Harris D Dela Cruz CS Possick JD. Pneumococcal vaccination strategies. an update and perspective. Ann Am Thorac Soc. (2016) 13:933–44. 10.1513/AnnalsATS.201511-778FR 27088424 PMC5461988

[56] Davey ME Duncan MJ. Enhanced biofilm formation and loss of capsule synthesis: deletion of a putative glycosyltransferase in *Porphyromonas gingivalis*. J Bacteriol. (2006) 188:5510–23. 10.1128/JB.01685-05 16855241 PMC1540017

[57] Laine ML Appelmelk BJ van Winkelhoff AJ. Novel polysaccharide capsular serotypes in *Porphyromonas gingivalis*. J Periodontal Res. (1996) 31:278–84. 10.1111/j.1600-0765.1996.tb00494.x 8814599

[58] Califano JV Schifferle RE Gunsolley JC Best AM Schenkein HA Tew JG. Antibody reactive with *Porphyromonas gingivalis* serotypes K1-6 in adult and generalized early-onset periodontitis. J Periodontol. (1999) 70:730–5. 10.1902/jop.1999.70.7.730 10440633

[59] Choi JI Schifferle RE Yoshimura F Kim BW. Capsular polysaccharide-fimbrial protein conjugate vaccine protects against *Porphyromonas gingivalis* infection in SCID mice reconstituted with human peripheral blood lymphocytes. Infect Immun. (1998) 66:391–3. 10.1128/IAI.66.1.391-393.1998 9423888 PMC107916

[60] Sun X Stefanetti G Berti F Kasper DL. Polysaccharide structure dictates mechanism of adaptive immune response to glycoconjugate vaccines. Proc Natl Acad Sci USA. (2019) 116:193–8. 10.1073/pnas.1816401115 30510007 PMC6320544

[61] Rappuoli R De Gregorio E Costantino P. On the mechanisms of conjugate vaccines. Proc Natl Acad Sci USA. (2019) 116:14–6. 10.1073/pnas.1819612116 30578318 PMC6320500

[62] Munoz FM Cramer JP Dekker CL Dudley MZ Graham BS Gurwith M. Vaccine-associated enhanced disease: case definition and guidelines for data collection, analysis, and presentation of immunization safety data. Vaccine. (2021) 39:3053–66. 10.1016/j.vaccine.2021.01.055 33637387 PMC7901381

[63] Mascola JR Mathieson BJ Zack PM Walker MC Halstead SB Burke DS. Summary report: workshop on the potential risks of antibody-dependent enhancement in human HIV vaccine trials. AIDS Res Hum Retroviruses. (1993) 9:1175–84. 10.1089/aid.1993.9.1175 7908211

[64] Sims TJ Schifferle RE Ali RW Skaug N Page RC. Immunoglobulin G response of periodontitis patients to *Porphyromonas gingivalis* capsular carbohydrate and lipopolysaccharide antigens. Oral Microbiol Immunol. (2001) 16:193–201. 10.1034/j.1399-302X.2001.160401.x 11442843

