# Supplementary figures and images for: A Porphyromonas gingivalis Capsule-Conjugate Vaccine Protects From Experimental Oral Bone Loss
Source: Front Oral Health. 2021 Jul 5;2:686402. doi: 10.3389/froh.2021.686402 (PMC8757777; doi:10.3389/froh.2021.686402)

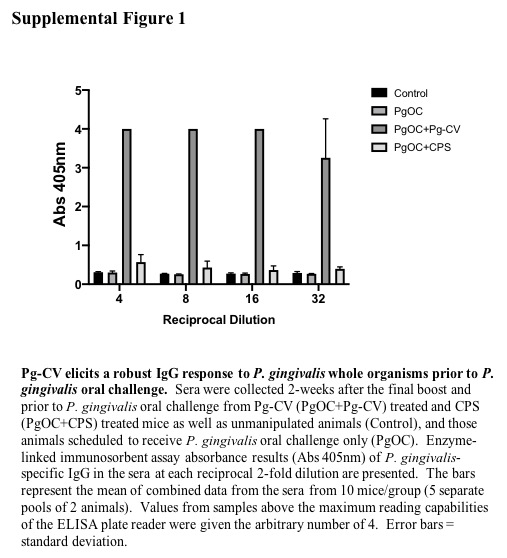

Supplement: Supplementary file 1 [file Image_1.JPEG]

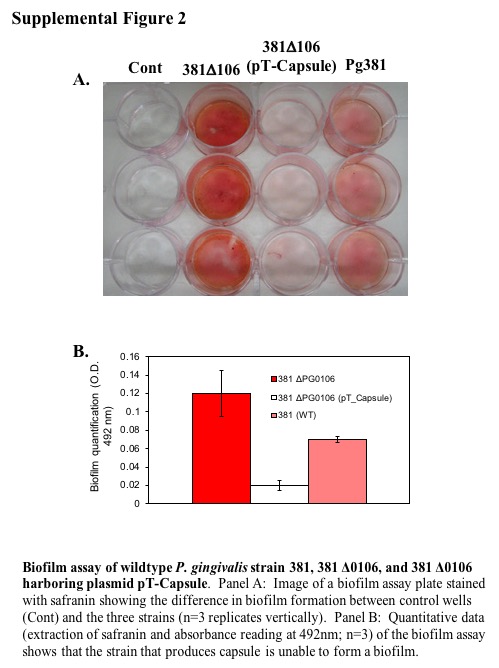

Supplement: Supplementary file 2 [file Image_2.JPEG]
